# DNA Markers to Discriminate *Cannabis sativa* L. ‘Cheungsam' with Low Tetrahydrocannabinol (THC) Content from Other South Korea Cultivars Based on the Nucleotide Sequences of *Tetrahydrocannabinolic Acid Synthase* and Putative *3-Ketoacyl-CoA Synthase* Genes

**DOI:** 10.1155/2019/8121796

**Published:** 2019-11-22

**Authors:** Eui Jeong Doh, Guemsan Lee, Yeong-Jin Yun, Lin-Woo Kang, Eun Soo Kim, Mi Young Lee, Seung-Eun Oh

**Affiliations:** ^1^Department of Biological Sciences, Konkuk University, Seoul 05029, Republic of Korea; ^2^Korea Hemp Institute, Konkuk University, Seoul 05029, Republic of Korea; ^3^Department of Herbology, College of Korean Medicine, Wonkwang University, Iksan 54538, Republic of Korea; ^4^Clinical Medicine Division, Korea Institute of Oriental Medicine, Daejeon 34054, Republic of Korea

## Abstract

*Cannabis sativa* L. has been utilized for a long time as a traditional herbal medicine in Korea. Dry fruits, achenes, each containing a single seed of *Cannabis*, are currently prescribed as *Ma In* (Cannabis Semen), a laxative. As each achene is enclosed by a bract, in which tetrahydrocannabinol (THC), the main psychological active compound in *Cannabis* is synthesized; achene is easily contaminated by THC from bract remnants. Therefore, it is safer to harvest achenes from *Cannabis* with a low THC content. Seeds of hemp, a low THC *Cannabis*, were recently classified as possible sources of new pharmacologically active compounds. Thus, a proper method to select appropriate *Cannabis* plants with low THC among cultivars in South Korea for medicinal purpose is necessary. As a result of cross-selection, *Cannabis* L. cultivar “Cheungsam” (CH) with the lowest THC content among cultivars cultivated in South Korea has been developed. In this study, we developed two DNA markers to reliably discriminate CH from other local cultivars with higher THC contents. We developed primer sets CHF3/CHR2 to amplify the 642 bp DNA marker of CH based on differences in the nucleotide sequences of the *THCA synthase* gene, which encodes a key enzyme in THC synthesis. We then developed a CHF1/CHR3 primer set to amplify the 401 bp DNA marker of CH based on the differences in both the content of very long chain fatty acids (VLCFs) and the sequence of the putative *3-ketoacyl-CoA synthase* (*KCS*) gene encoding enzymes synthesizing VLCFs among local cultivars.

## 1. Introduction

*Cannabis sativa* L. has been cultivated for a long time for the supply of fibers, foods, and medicines [1]. Owing to selective breeding for centuries, over 700 different varieties and cultivars of *C. sativa* have been described and many more are thought to exist [2]. Varieties and cultivars of *Cannabis* are generally classified into two groups based on their purpose or tetrahydrocannabinol (THC) content (the primary psychological active component in *Cannabis*) and not on taxonomy [3]. The industrial nondrug *Cannabis*, known as hemp, has been cultivated for its fibers and seeds for thousands of years [4, 5], whereas the drug type, known as marijuana or hashish, is cultivated to obtain the intoxicant compound [6]. The THC content in the drug type is 1–3.7% (dry weight) [6], whereas in hemp, it is less than 0.2% [7] or 0.35% [8].

Achenes of *C. sativa* L. are currently prescribed as *Ma In* (Cannabis Semen; *Huomaren* (Cannabis Fructus) in Chinese), an herbal medicine used in Korea [9–11]. *Ma In* is used as a laxative; it stimulates the intestinal mucosa to excrete waste products through the large intestine and is especially formulated for old and pregnant individuals [12]. Each achene is enclosed by a persistent floral bract, which has a large number of glandular trichomes synthesizing various cannabinoids, including THC. The THC levels in *Cannabis* are highest in the bracts [13]. Therefore, achenes are easily contaminated by THC from bract remnants, confirmed using monoclonal antibodies against THC [14]. Achenes are suitable sources of *Ma In* that could be harvested from low THC *Cannabis* varieties or cultivars. Recently, various pharmacologically active compounds have been uncovered in seeds of low THC *Cannabis* plants, which have been classified as hemp; low THC *Cannabis* achenes are evaluated as sources of new compounds, which have health benefits [15]. Therefore, the selection of ideal *Cannabis* plants cultivated in South Korea is necessary to supply the proper sources of *Ma In* and develop new biologically active compounds.

Owing to cross fertilization, a new *Cannabis* cultivar, ‘Cheungsam' (CH), which has a THC content of 0.34%, has been developed in Korea [16]. Cheungsam was selected because it contained low THC concentrations and exhibited the best agronomic characteristics under South Korean environmental conditions among F_1_s of Korean local cultivars with high levels of THC (around 1.7%) and introduced low THC accessions (0.08–0.28%) from the Center for Plant Breeding and Reproduction Research (CPRO) in the Netherlands [16, 17]. Cheungsam is currently the lowest THC *Cannabis* among cultivars cultivated in South Korea.

Kojoma et al. [18] suggested that the substitution of amino acids caused by variations in nucleotides could result in an intense reduction in the activities of tetrahydrocannabinolic acid (THCA) synthases, a key enzyme used to synthesize THC and the level of THC in *Cannabis*. Doh [19] found variations in the composition and fatty acid content of achenes, especially in very long chain fatty acids (VLCFAs) synthesized by VLCFA elongase (3-ketoacyl-CoA synthase; KCS) among Korean cultivars. In this study, we developed two DNA markers to discriminate, indubitably, CH from other local South Korean cultivars with high THC content based on differences in the nucleotide sequences of the *THCA synthase* and putative *KCS* genes encoding key enzymes in the synthesis of VLCFAs for the supply of *Cannabis* achenes or seeds appropriate for medicinal use.

## 2. Materials and Methods

### 2.1. Plant Materials

Achenes of CH and local *Cannabis* cultivars from South Korea were collected from provinces, such as Dangjin (central western province), Boseung (south western province), Jecheon (central province), Jeogseon, and Andong (central eastern provinces) (Table 1). As the same cultivar was found in both Jungseon and Andong provinces, they will hereafter be referred to as “JU.” The samples listed in Table 1 are 1 g of each cultivar randomly selected from harvested seeds. Achenes used in this study were deposited as specimens at the department of herbology in the College of Korea Medicine, Wonkwang University.

### 2.2. Preparation of Genomic DNA

The genomic DNA of each sample was extracted using the NucleoSpin® Plant II (Macherey-Nagel, Duerern, Germany) according to the manufacturer's instructions.

### 2.3. Polymerase Chain Reaction (PCR) Amplification

#### 2.3.1. Amplification of THCA Gene

The amplifications of *THCA* synthase genes by PCR were conducted using a T-personal cycler (Biometra, Goettingen, Germany) according to the protocol by Sirikantaramas et al. [20]. Briefly, 1.2 pmol of forward primer (5-TGAAGAAAAAAAATGAATTGCTCAGCATTTTCC-3′) and reverse primer (5′-TCTATTTAAAGATAATTAATGATGATGCGGTGG-3′), 1 U Taq polymerase (ABgene, Epson, UK), and 20 ng of genomic DNA extracted from each sample were used. The 35-cycle PCR process consisted of pre-denaturation for 5 min at 95°C, denaturation for 30 s at 95°C, annealing for 30 s at 58°C, extension for 1 min at 72°C, and a final reaction step for 7 min at 72°C. The amplified PCR product was separated on 1.5% agarose gel electrophoresis after staining with Safe-white™ (ABM, Canada) and analyzed using MyImage (Seoulin Biotechnology, Seoul, Korea).

#### 2.3.2. Amplification of Putative *KCS* Gene

For the PCR amplification of putative *KCS* genes in *C. sativa*, forward primer HKCS F (5′-ATGACGTCCATTAACGTAAAGCTCC-3′) and reverse primer HKCS R (5′-TTAGGACCGACCG TTTTGGGC-3′) were designed based on the nucleotide sequences of *KCS* genes deposited in the NCBI GenBank. Approximately 2.4 pmol of both primers, 1 U Taq polymerase (ABgene, Epson, UK), and 20 ng of genomic DNA were used for PCR amplification. The 35-cycle PCR process consisted of pre-denaturation for 5 min at 95°C, denaturation for 30 s at 95°C, annealing for 30 s at 57°C, extension for 40 s at 72°C, and a final extension for 5 min at 72°C. The amplified PCR product was separated on 1.5% agarose gel electrophoresis after staining with Safe-white™ (ABM, Canada) and analyzed using MyImage (Seoulin Biotechnology, Seoul, Korea).

#### 2.3.3. Amplification of DNA Markers

For the amplification of a 642 bp DNA marker for the discrimination of CH from other Korean local cultivars based on the nucleotide sequences of *THCA* genes, forward primer CHF3 (5′-TAGTACTCATGACTCACTTCAG-3′) and reverse primer CHR2 (5′-GTGTAATTATTAGGACTCG-CAG-3′) were used. The CHF3/ISR (5′-TCCACCATGAAAAATTGAAGA-3′) primer set was used to amplify 100 bp size internal standards (IS) to evaluate the PCR process. For the amplification of the 401 bp sized DNA marker for cultivar CH based on the nucleotide sequences of *putative KCS* genes, forward primer CHF1 (5′-TAGCTCATTCAGTCCGACGC-3′) and reverse primer CHR3 (5′-CGATTGTGCCTCTATCGG-3′) were used. The CHF1/ISR2 (5′-GAATGAGATTAAGCCGGC-3′) primer set was used as IS to amplify the 121 bp product. Briefly, 2.4 pmol of the CHF3/CHR2/IS R and CHF1/CHR3/ISR2 primer sets were used with 1 U Taq polymerase (ABgene, Epson, UK) and 20 ng of genomic DNA. The 23-cycle PCR amplification process of both DNA markers included pre-denaturation for 5 min at 95°C, denaturation for 30 s at 95°C, annealing for 30 s at 54°C, and an extension process for 1 min at 72°C.

#### 2.3.4. Determination of Nucleotide Sequence-Amplified Products by PCR

PCR products separated on the agarose gel were cloned using the pGEM®-T Vector System I (Promega, Madison, WI, USA). The nucleotide sequences of cloned PCR products were determined by the Sanger sequencing method [21].

#### 2.3.5. Alignment of Nucleotide Sequences and Construction of the Dendrogram

Nucleotide sequences were aligned using the Clustal W multiple sequence alignment program in Bioedit v.7.0.9 (http://mbio.ncsu.edu/BioEdit/page2.html). A THCA synthase dendrogram was constructed using DNADist in Bioedit. As an outer-group of a dendrogram, nucleotide sequences of genes encoding CBDA synthase of *C. sativa* (accession number AB292682.1) and berberine bridge enzymes of *Eschscholtzia californica* (AF005655.1) were used.

## 3. Results

### 3.1. Discrimination of CH from Korean Local Cultivars Based on Nucleotide Sequences of THC Synthase Genes

**Figure 1:**
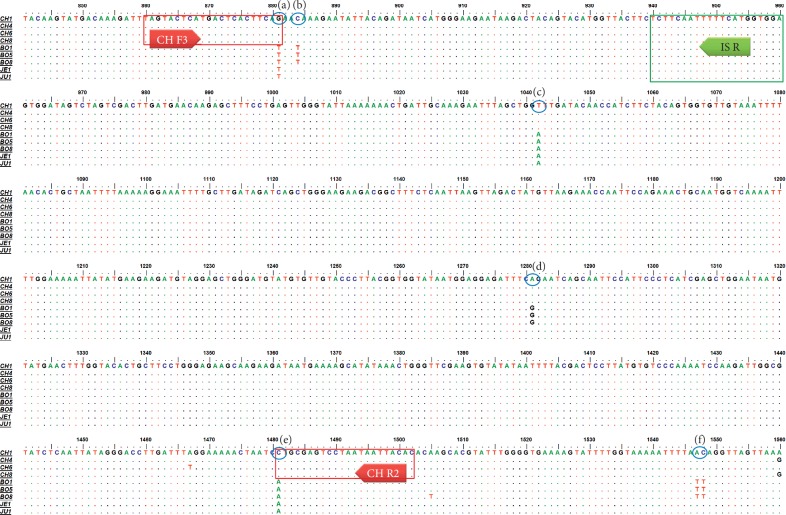
Multiple alignment of the partial nucleotide sequences of *tetrahydrocannabinolic acid* (*THCA*) *synthase* genes from samples listed in Table 1. The dots indicate the consensus nucleotide; bold arrows indicate the primers used to amplify DNA markers of CH and the internal standard; the boxes represent nucleotide sequences and the positions of primers CHF3, CHR2, and ISR. (a)–(c) represent residues that might encode the regions located in the substrate-binding pocket beside to the FAD cofactor, and (d)–(f) represent residues that might encode the regions located on the surface of THCA synthase. The regions encoded by residues were predicted on the basis of the uncovered crystal structure of THCA synthase (PDB ID 3VTE) from *Cannabis sativa* L.

**Figure 2:**
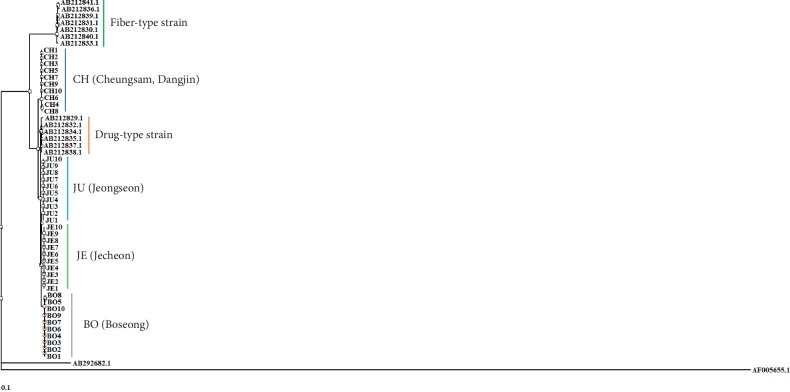
Dendrogram based on the nucleotide sequences of *tetrahydrocannabinolic acid* (*THCA*) *synthase* genes of samples listed in Table 1 and deposited in NCBI GenBank. The deposited nucleotide sequences of *THC synthase* in drug-type *Cannabis* (AB212829.1, AB212832.1, AB212834.1, AB212835.1, AB2128837.1, and AB212838.1) and hemp (AB212830.1, AB212831.1, AB212833.1, AB212836.1, AB212839.4, AB212840.1, and AB212841.1) were used to construct the dendrogram. As outer-groups of a dendrogram, nucleotide sequences of genes encoding *CBDA synthase* of *C. sativa* (AB292682.1) and berberine bridge enzyme of *E. californica* (AF005655.1) were used.

**Figure 3:**
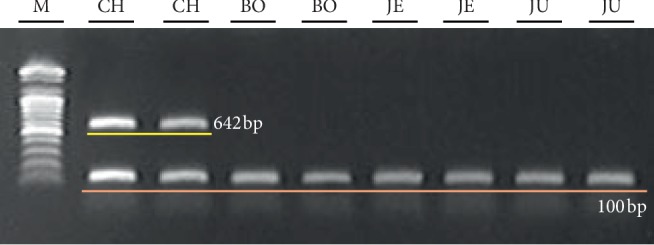
Polymerase chain reaction products of the primer sets, CHF3/CHR2 and CHF3/ISR, presented in Figure 1 from randomly chosen samples in Table 1 for the discrimination of “Cheungsam” cultivar. M: 100 bp ladder.

To distinguish CH, which is proper *Cannabis*, from *Cannabis* plants cultivated in South Korea for medicinal purposes, we developed DNA markers of CH. First, we tested whether CH could be efficiently separated from other Korean local cultivars with high THC content based on differences in nucleotide sequences of the *THCA synthase* gene in 40 samples (Table 1). Using primers designed by Sirikantaramas et al. [20], we amplified and determined the nucleotide sequences of 1,665 bp *THCA synthase* genes in CH and other cultivars (data not shown). Determined sequences were deposited in the NCBI GenBank (MN422084-MN422092). As shown in Figure 1, there were differences in the nucleotide sequences of *THCA synthase* genes among examined cultivars and within each cultivar. We detected 20 nucleotide variations between CH and BO, and eight nucleotide variations between JE and JU. There was no nucleotide variation within cultivar JE or JU. In contrast, two nucleotide variations were detected in two samples (B5 and B8) of BO and three nucleotide variations were detected in three samples (CH4, CH6, and CH8) of CH (Figure 1). To confirm whether CH could be differentiated from other cultivars based on differences in the sequences of *THCA synthase* genes despite the presence of nucleotide variations within CH or BO, we constructed a dendrogram based on the nucleotide sequences of *THCA synthase* genes (Figure 2). The nucleotide sequences of *THCA synthase* genes determined in this study and deposited in NCBI GenBank, which were isolated in *Cannabis* varieties or cultivars and classified into drug or nondrug types, were used to construct the dendrogram. The group composed of CH samples significantly differed from other groups consisting of local cultivars on the dendrogram (Figure 2). Therefore, we expected to be able to develop a DNA marker to distinguish CH from local cultivars based on differences in the nucleotide sequences of *THCA synthase* genes. We designed primer set CHF3/CHR2 based on variations in the 893^rd^ and 1493^rd^ nucleotides to amplify 642 bp DNA markers, which appeared solely in the CH samples (Figure 1). We also designed the primer set CHF3/ISR to amplify the 100 bp size internal standard (IS) and confirm the PCR amplification (Figure 2). As shown in Figure 3, 642 bp PCR products appeared uniquely, as expected, in randomly chosen CH samples listed in Table 1.

### 3.2. Amplification of Putative *KCS* Genes in CH and Korean Local Cultivars

**Figure 4:**
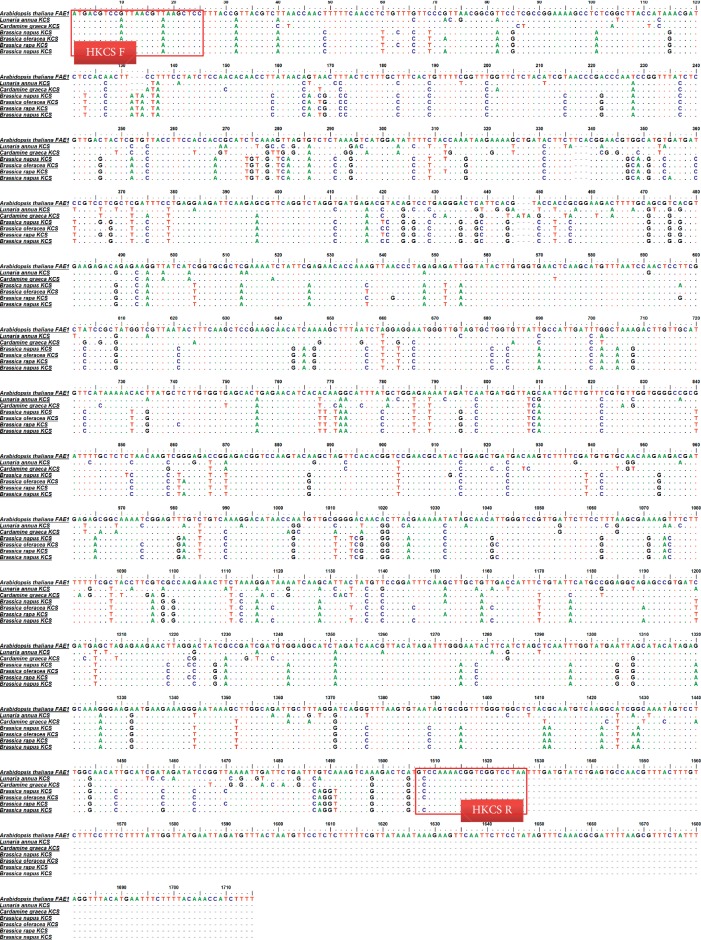
Multiple alignment of the nucleotide sequences of *3-ketoacyl-CoA synthase* (*KCS*) genes deposited in NCBI GenBank. The nucleotide sequences of *KCS* genes in *Arabidopsis thaliana* (EU29142.1), *Brassica oleracea (*AF490460.1), *Brassica napus* (AF490459.1 and AF490462.1), *Brassica rapa* (AF490461.1), *Cardamine graeca* (EU871788.1), and *Lunaria annua* (EU871787.1) were aligned. The dots indicate the consensus nucleotide; bold arrows indicate the primers to amplify putative KCS synthase genes. The boxes represent nucleotide sequences as well as the positions of primers.

**Figure 5:**
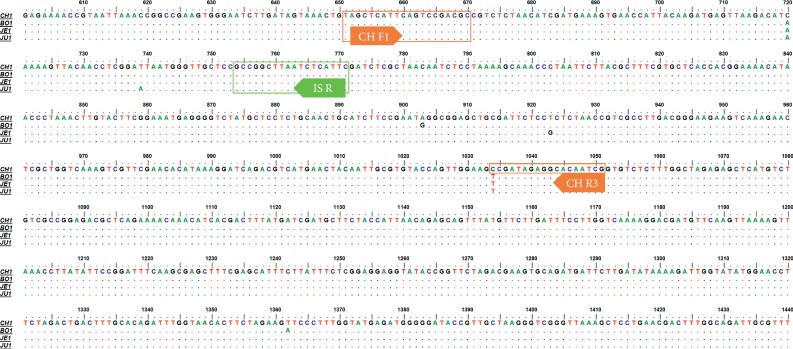
Multiple alignment of the nucleotide sequences of partial putative *3-ketoacyl-CoA synthase* (*KCS*) *synthase* genes amplified by the HKCS F/HKCS R primer set presented in Figure 4 from samples in Table 1. The dots indicate the consensus nucleotide; bold arrows indicate the primers to amplify DNA markers of CH and the internal standard; the boxes represent nucleotide sequences as well as the positions of primers CHF1, CHR3, and ISR.

We uncovered variations in the composition and concentration of fatty acids in seeds contained in achenes among CH and other local Korean cultivars, especially in VLCFs [19]. Therefore, we inferred that the variations in the nucleotide sequences of *KCS* genes encoding key enzymes of VLCFA biosynthesis could be another criterion used to select appropriate *Cannabis* from among South Korean cultivars for the medicinal applications. To the best of our knowledge, *KCS* genes participating in the synthesis of VLCFAs in seeds of *Cannabis sativa* L. have not been isolated as of yet. We aligned and compared the nucleotide sequences of seven *KCS* genes isolated from various plants, such as *Arabidopsis* and *Brassica*, deposited in the NCBI GenBank to determine the conserved regions of *KCS* genes, in which the consensus in the nucleotides among plants is shown (Figure 4). We also designed the primer set HKCS R/HKCS to amplify 1,563 bp regions in seven *KCS* genes (Figure 4). We amplified putative *KCS* genes in CH and local cultivars using this primer set, determined the nucleotide sequences of these PCR products, and deposited the sequences in the NCBI GenBank (MN422080-MN422083). There were no variations in the sequences of amplified PCR products within CH and other cultivars. Among cultivars, four nucleotide variations between CH and BO, and two nucleotide variations between JE and JU were detected. The partial nucleotide sequence of amplified PCR products of CH and other local cultivars is represented in Figure 5.

### 3.3. Discrimination of CH from Korean Local Cultivars Based on the Nucleotide Sequences of Putative *KCS* Genes

**Figure 6:**
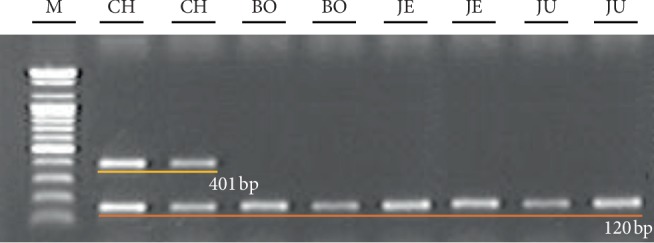
Polymerase chain reaction products of the primer sets, CH F1/CH R3 and CH F1/IS R2, presented in Figure 5 from randomly chosen samples in Table 1 for the discrimination of “Cheungsam” cultivar. M: 100 bp ladder.

To distinguish CH definitively from other South Korean local cultivars, we developed another DNA marker of CH based on the difference in the sequences of amplified putative *KCS* genes among cultivars. We designed the primer set CHF1/CHR3 to amplify the 401 bp DNA marker, which appeared only in samples of CH based on the substitution of C at the 1034^th^ nucleotide of the amplified PCR product in CH to T in local cultivars (Figure 5). We also designed the primer set CHF1/ISR, which amplified the 121 bp internal standard for confirmation of the PCR process (Figure 5). As shown in Figure 6, 401 bp PCR products were amplified only in CH samples, as expected.

## 4. Discussion

### 4.1. CH Is Distinguishable from Korean Local Cultivars by the Differences in the Sequence of the THCA Synthase Gene

The chemical features of *Cannabis*, specifically the THC content, are used to classify varieties and cultivars into drug and nondrug types (hemp) [7, 18]. However, the content of THC varies depending on the tissue or organ type [22]. It is also dependent on the developmental stage of the plant and environmental conditions, such as light and nutrients, under which it develops [23–25]. Moreover, long-term storage of *Cannabis* results in partial degradation of THC by light and oxygen [26]. In the former USSR and current Europe, 0.2% THC was the maximum permitted content for *Cannabis* to be classified as hemp [7, 27]. Apropos of this, currently, 0.3% and 0.35% THC are the maximum permitted content in Canada and New Zealand, respectively [8, 27]. In contrast, the relative content of THC to cannabidiol (CBD) (THC/CBD ratio), both of which are converted from the same precursor, cannabigerol, is believed to remain constant throughout all the developmental stages, relatively unaffected by environmental factors [25, 28]. When the ratio of THC/CBD is more than 1, *Cannabis* is classified as a drug type [7]. Our study was restricted to achenes, where THC and/or CBD was not detected unless and until contamination derived from bracts occurred [29]. Therefore, we were not able to compare the THC and THC/CBD ratio in CH and the other examined local cultivars. According to a report by Moon et al. [16] who developed CH, the THC and CBD levels in CH were 0.34% and 1.34%, respectively. Therefore, CH is on the border of being a drug and nondrug based on its absolute THC content. However, by the criterion of the THC: CBD ratio, CH is classified as hemp. Hillig and Mahlberg [30] and Hilling [31] classified landraces cultivated in eastern Asia, including Korea, into one putative taxon, *C. indica* biotype based on the two-species concept of *Cannabis* taxonomy or one of putative taxa comprising the *C. indica* gene pool based on the polytypic concept of *Cannabis* composed 3 species (*C. sativa*, *C. indica*, and *C. ruderalis*). Hillig and Mahlberg [30] showed that average THC levels were significantly higher in *C. indica* than in *C. sativa*. According to these results, almost all cultivars in South Korea could be drug-type *Cannabis* except CH. This possibility was further supported by the dendrogram presented in Figure 2. Cultivars examined in this study, excepting CH, were classified into subgroups of a drug-type group.

Seeds of hemp have been sources of essential amino acids and fatty acids for a long time [32]. However, the diverse pharmacological activities of hemp seeds in relation to human health, such as immunomodulatory effects, cardiovascular benefits, and anti-neuroinflammatory activities, were discovered recently [6, 32, 33]. Hemp is now considered a source of new bioactive compounds [15]. Therefore, CH might be an excellent source of not only *Ma In* but also for the development of new pharmacological compounds among cultivars examined from South Korea.

Although various DNA profiling methods, such as ITS and *trnL*–*F* intergenic spacer, have been applied to distinguish between CH and other cultivars, we were unable to find an effective method (data not shown).

Kojoma et al. suggested that types of *Cannabis* plants could be determined by the DNA polymorphism in *THCA synthase* gene [18]. A single nucleotide polymorphism (SNP) assay for the discrimination of drug from nondrug type *Cannabis* [34] and a DNA marker to predict THC content were developed [22] based on that suggestion.

Based on the uncovered crystal structure of THCA synthase of *Cannabis sativa* L. (PDB ID 3VTE), we predicted that residues which showed variations in the nucleotide sequences among cultivars especially marked as (a)–(c) in Figure 1 might encode region(s) located in the substrate binding pocket next to FAD cofactor. Other residues such as (e)–(f) might encode region(s) on the surface of THCA synthase. Therefore, variations in the nucleotide in these residues could affect the activity of THCA synthase in cultivar “CH” and finally decreased the content of THC. In this study, we applied variation in the nucleotide sequences of both residues (a) and (f) to design primers (Figure 1) to amplify 642 bp DNA markers to select “CH” (Figure 3)

### 4.2. Discrimination of CH from Korean Local Cultivars Based on the Differences in the Sequences of Putative *KCS* Gene

Doh [19] examined fatty acids in seeds of South Korea *Cannabis* cultivars. The main fatty acids were VLCFAs, such as C18 : 1, C18 : 2, and C18 : 3, as well as LCFAs (long chain fatty acids) such as C16 : 0 as reported previously [35–37]. Other VLCFAs, such as C20 : 0 and C22 : 2, were detected as minor fatty acids. Doh (2012) also found variations in the content and amount of fatty acids especially with respect to minor VLCFAs between CH and other local cultivars (data not shown).

The biosynthesis of VLCFs is catalyzed by acyl-CoA elongase, a membrane-bound enzyme complex composed of 3-ketoacyl-CoA synthase (KCS), 3-ketoacyl-CoA reductase (KCR), 3-hydroacyl-CoA dehydratase, and trans-2,3-enoyl-CoA reductase (ECR) [38]. The initial and rate-limiting step in fatty acid elongation is catalyzed by KCS. *FAE1*(*KCS19*), a *KCS* gene, which is believed to participate in the biosynthesis of C22 : 1 fatty acid in seeds, was first isolated in *A. thaliana* [39, 40]. Subsequently, 21 *KCS* genes, including *KCS2*, involved in the biosynthesis of specific VLCFAs, a precursor of aliphatic suberin in roots and seeds, were isolated in *A. thaliana* [41]. Therefore, we speculated that the amount and/or composition of VLCFA in seeds of *Cannabis* might, at least in part, be determined by the activity of specific *KCS* genes. The activity of KCS may be influenced by DNA polymorphism in *KCS* genes, and this DNA polymorphism could be used to distinguish CH from other local cultivars for the supply of sources of *Ma In* and developing new compounds.

As mentioned, it is well known that KCS are encoded by the multigene family. The number of members is increased up to 30 in divergent angiosperm species [42]. Therefore, for the amplification of putative *KCS* gene(s) participating in the synthesis of VLCFA in seeds of *Cannabis* in CH and other Korean cultivars by PCR, we compared the nucleotide sequences of seven *KCS* genes such as *FAE1* in *Arabidopsis* to analyze the presence of variables and/or consensus regions in the nucleotide sequences among *KCS* (Figure 4). The nucleotide sequences of *KCS* genes in *Cardamine graeca* and *Lunaria annua*, in which nervonic acid (C24 : 1) is synthesized in seeds [43, 44] and in genus *Brassica* containing erucic acid (C22 : 1) as major fatty acids in seed oils [45], were also compared. As shown in Figure 5, regions such as those encoding N terminal (from 1^st^ to 39^th^ nucleotide) and the middle of the sequence (from 556^th^ to 596^th^ nucleotide, from 622^nd^ to 642^nd^ nucleotide, and from 1, 252^nd^ to 1, 274^th^ nucleotide) of the *KCS* genes were well conserved. The 1, 509^th^ to 1, 527^th^ nucleotide of *KCS* genes were highly conserved in the region encoding the C terminal. Based on the consensus nucleotide sequences encoding N and C terminals in examined *KCS* genes, we designed the primer sets, HKCS F/HKCS R, to amplify 1,563 bp size putative *KCS* genes in examined *Cannabis* plants (Figure 4). When we compared the nucleotide sequences of those PCR products with sequences deposited in NCBI, *FAE1*, and *KCS* in *Arabidopsis* (U29142.1, AY096568.1, AY074285.1, AF053345.1, and AK226331.1) and *KCS* in *Brassica* (AY642539.1, XM 013749750.1, NM 001316053.1, and AY642539.1), 82–87% and 80–85% sequence identities, respectively, were observed. Further studies are necessary to confirm that the amplified products in this study are the expected *KCS* genes that participate in the synthesis of VLCFAs in *Cannabis* seeds. As presented in Figure 5, there was variation in the nucleotide sequences among amplified putative *KCS* genes in CH and other local cultivars used to design the CHF1/CHR3 primer set to amplify 401 bp DNA markers to discriminate CH from other local cultivars (Figure 6). When these two developed DNA markers in this study are combined, we can select proper CH more definitively to supply resources for medicinal purposes among *Cannabis* cultivated in South Korea.

## 5. Conclusion

*Cannabis sativa* L. ‘Cheungsam' (CH), in which the THC content is the lowest among examined cultivars in South Korea, is the most appropriate *Cannabis* plant for the supply of achenes as herbal medicine, *Ma In*, and sources for developing new pharmacological compounds in seeds. In this study, we developed two specific DNA markers of CH to distinguish it from other local cultivars with higher THC contents. A 642 bp DNA marker of CH based on the differences in the nucleotide sequences of genes encoding THCA synthase, a key enzyme for THC synthesis, was amplified by the primer set CHF3/CHR2 designed in this study. The other 401 bp DNA maker of CH based on differences in the nucleotide sequences of putative 3-ketoacyl-CoA synthase (KCS), which plays a role in the synthesis of VLCFA, was amplified by the primer set CHF1/CHR3.

## Figures and Tables

**Table 1:** The list of *C. sativa* achenes used in this study.

Sample	Locality	Date of collection	Voucher no.
CH1	Dangjin	2009.7	WCSCH01
CH2		2009.7	WCSCH02
CH3		2009.7	WCSCH03
CH4		2009.7	WCSCH04
CH5		2009.7	WCSCH05
CH6		2009.7	WCSCH06
CH7		2008.7	WCSCH07
CH8		2008.7	WCSCH08
CH9		2008.7	WCSCH09
CH10		2008.7	WCSCH10

BO1	Boseung	2009.7	WCSBO01
BO2		2009.7	WCSBO02
BO3		2009.7	WCSBO03
BO4		2009.7	WCSBO04
BO5		2009.7	WCSBO05
BO6		2009.7	WCSBO06
BO7		2008.7	WCSBO07
BO8		2008.7	WCSBO08
BO9		2008.7	WCSBO09
BO10		2008.7	WCSBO10

JU1	Jeongseon/Andong	2008.7	WCSJU01
JU2		2008.7	WCSJU02
JU3		2008.7	WCSJU03
JU4		2008.7	WCSJU04
JU5		2008.7	WCSJU05
JU6		2008.7	WCSJU06
JU7		2008.7	WCSJU07
JU8		2008.7	WCSJU08
JU9		2008.7	WCSJU09
JU10		2008.7	WCSJU10

JE1	Jecheon	2008.7	WCSJE01
JE2		2008.7	WCSJE02
JE3		2008.7	WCSJE03
JE4		2008.7	WCSJE04
JE5		2008.7	WCSJE05
JE6		2008.7	WCSJE06
JE7		2008.7	WCSJE07
JE8		2008.7	WCSJE08
JE9		2008.7	WCSJE09
JE10		2008.7	WCSJE10

## Data Availability

The nucleotide sequence data, besides those deposited in the NCBI GenBank, that support the findings of this study are available from the first or corresponding author upon request.
